# CYP2S1 and CYP2W1 expression is associated with patient survival in breast cancer

**DOI:** 10.1002/cjp2.291

**Published:** 2022-07-28

**Authors:** Radhika Aiyappa‐Maudsley, Sarah J Storr, Emad A Rakha, Andrew R Green, Ian O Ellis, Stewart G Martin

**Affiliations:** ^1^ Nottingham Breast Cancer Research Centre, School of Medicine, Biodiscovery Institute University of Nottingham, University Park Nottingham UK; ^2^ Present address: Cancer Research Centre, Department of Molecular and Clinical Cancer Medicine University of Liverpool, William Henry Duncan Building Liverpool UK

**Keywords:** breast cancer, cytochrome P450, metabolism, prognosis, immunohistochemistry

## Abstract

The cytochrome P450 family of enzymes metabolise a wide range of compounds and play important roles in breast cancer pathogenesis due to their involvement in estrogen metabolism and the production of carcinogenic metabolites during this process. The orphan CYPs, CYP2S1, and CYP2W1 are reportedly upregulated in breast cancer. However, their expression and association with clinicopathological and survival parameters have not been previously assessed in a large cohort of breast cancers. Protein expression of CYP2S1 and CYP2W1 was assessed in early‐stage invasive breast cancers (*n* = 1,426) using immunohistochemistry and correlated with various clinicopathological parameters and survival. mRNA expression of *CYP2S1* and *CYP2W1* was also assessed in the Molecular Taxonomy of Breast Cancer International Consortium (METABRIC) cohort. Low nuclear and cytoplasmic CYP2S1 was significantly associated with high‐grade tumours (*p* ≤ 0.009), intermediate Nottingham prognostic index (NPI) group (*p* ≤ 0.025), high mitotic frequency (*p* ≤ 0.002), human epidermal growth factor receptor 2 (HER2)‐negative disease (*p* ≤ 0.011), and ductal carcinoma (*p* ≤ 0.022). Cytoplasmic CYP2S1 was additionally associated with patients ≥50 years (*p* < 0.001), estrogen receptor (ER)‐positive tumours (*p* = 0.011), and high nuclear pleomorphism (*p* = 0.003). Low cytoplasmic CYP2W1 was significantly associated with patients ≥50 years (*p* = 0.002), HER2‐negative disease (*p* = 0.003), intermediate NPI (*p* = 0.013), and mitosis (*p* = 0.009). Low cytoplasmic CYP2S1 was significantly associated with adverse breast cancer specific survival (*p* = 0.034), which remained so in multivariate analysis (hazard ratio [HR]: 0.639; 95% confidence interval [CI]: 0.483–0.846; *p* = 0.002). Low nuclear CYP2W1 was significantly associated with adverse breast cancer specific survival (*p* = 0.012), with significance also maintained in multivariate analysis (HR: 0.677; 95% CI: 0.510–0.898; *p* = 0.007). No associations with survival were observed in the METABRIC cohort. CYP2S1 and CYP2W1 are associated with patient survival in breast cancer and may be important prognostic biomarkers.

## Introduction

The cytochrome P450 (CYP) family of isoenzymes are involved in the metabolism of diverse endogenous compounds (e.g. vitamins A and D, fatty acids, and eicosanoids) and detoxification of exogenous agents (e.g. natural products, drugs, and carcinogens) [1, 2, 3]. They can activate or inactivate several pre‐carcinogenic compounds as well as chemotherapeutic drugs, which links them to cancer initiation and progression [4]. They are important in the synthesis of hormones such as estrogen and testosterone [1, 5]. To date, around 57 CYP genes have been identified in total, of which 13 have gained ‘orphan’ status as their metabolic functions remain unclear [6].

Cytochrome P450 2S1 (CYP2S1) is one such ‘orphan’ CYP, and is particularly expressed in epithelial cells of tissues which are exposed to the environment such as the skin, respiratory, gastrointestinal, and urinary tracts. They are also commonly expressed in cancers of epithelial origin [7]. CYP2S1 is associated primarily with the synthesis and metabolism of lipids including prostaglandins (PGE_2_) and retinoids [8]. Immunohistochemical analysis of CYP2S1 conducted in 170 breast cancers reported that high expression was associated with shorter patient survival, although this was not confirmed in multivariate analysis [9]. Another CYP enzyme with ‘orphan’ status is cytochrome P450 2W1 (CYP2W1), which is endogenously expressed in foetal colon and has minimal expression in normal tissues [10]. CYP2W1 demonstrates tumour‐specific expression, especially in epithelial tumours [11, 12]. The mRNA expression of *CYP2W1* is reportedly upregulated in breast cancer and influences response to neoadjuvant chemotherapy (NACT) [13]. CYP2W1 has also been reported to be an independent prognostic marker in stage II and III colon cancers [14]; however, no studies have reported its prognostic significance in breast cancer.

The CYP enzymes are associated with the onset of several hormone‐dependent cancers, mainly through their involvement in estrogen metabolism. They eliminate estrogens from the body by converting them into inactive metabolites in the liver that are subsequently excreted [15, 16]. CYPs catalyse the first step in the metabolism of estrogens, i.e. hydroxylation of estrogens into 2‐hydroxy (2‐OHE_2_) and 4‐hydroxyestradiol (4‐OHE_2_) metabolites [17, 18]. The hydroxylated products can undergo redox cycling to form quinones and semiquinones that react with DNA to generate highly mutagenic sites [19, 20]. Reactive oxygen species (ROS) such as hydrogen peroxide (H_2_O_2_) and hydroxyl radicals (OH˙) that are generated during redox cycling can cause oxidative DNA damage and lipid peroxidation. All of these can further stimulate estrogen metabolism, leading to additional semiquinone–quinone cycling, ROS production, and DNA damage, all of which may contribute to the initiation of breast, prostate, and other cancers [19]. The CYP enzymes are also implicated in the metabolism of tamoxifen [21] and bioactivation of lapatinib [22], used in the treatment of hormone receptor (HR) and human epidermal growth factor receptor 2 (HER2)‐positive breast cancers respectively. The CYP enzymes involved in the metabolism of breast cancer chemotherapeutic agents have been reviewed elsewhere [23]. CYP2S1 and CYP2W1 are also involved in the metabolism of benzothiazoles [24], and in the bioreductive activation of certain hypoxia‐activated prodrugs (HAPs), such as AQ4N [24]. They catalyse the hypoxic reduction of AQ4N to AQ4, at a rate of approximately 12 mole of substrate per mole of enzyme per minute – the highest turnover rates compared to the previously identified CYP3A4 and inducible nitric oxide synthase [24]. Therefore, identifying CYP2S1 and CYP2W1 expression in patient tumours may help in the selection of individuals that could potentially benefit from treatment with chemotherapeutic agents that require these enzymes for metabolism. Although the prognostic values of CYP2S1 and CYP2W1 have been studied in other cancers, such as colon, there is little information regarding the prognostic value of CYP2S1 and CYP2W1 in breast cancers. The aim of the study was to investigate the protein expression of CYP2S1 and CYP2W1 in a large independent cohort of breast cancer patient tumour samples by immunohistochemistry. *CYP2S1* and *CYP2W1* mRNA expression was also evaluated in a separate cohort of patients, the Molecular Taxonomy of Breast Cancer International Consortium (METABRIC) cohort. CYP2S1 and CYP2W1 protein/mRNA were correlated to various clinicopathological parameters and survival, in both the Nottingham and METABRIC cohorts respectively.

## Materials and methods

### Ethics approval

This study was approved by the Nottingham Research Ethics Committee 2 under the title ‘Development of a molecular genetic classification of breast cancer’ (REC202313) and by North West – Greater Manchester Central Research Ethics Committee under the title ‘Nottingham Health Science Biobank (NHSB)’ (15/NW/0685). All procedures performed in studies involving human participants were in accordance with the ethical standards of the institutional and/or national research committee and with the 1964 Helsinki Declaration and its later amendments or comparable ethical standards.

### Nottingham cohort (IHC assessments)

This study is reported according to REMARK (reporting recommendations for tumour marker prognostic studies) criteria [25]. A total of 1,426 primary operable early invasive breast cancer patients, treated surgically in Nottingham City Hospital between 1998 and 2006, were included. All patients were treated in a standardised manner, by mastectomy or wide local excision, as decided by disease characteristics or patient choice, followed by radiotherapy if indicated. Breast cancer specific survival (BCSS) was calculated as the time‐interval (in months) between primary surgery and death resulting from breast cancer. Patients were administered systemic adjuvant therapy (hormonal therapy [HT] and cyclophosphamide, methotrexate, and 5‐flourouracil [CMF]), depending on tumour prognostic and predictive factors including the Nottingham prognostic index (NPI), lymph node status, menopausal status, and estrogen receptor (ER) status. Patients stratified into the good prognostic group (NPI ≤ 3.4) did not receive adjuvant therapy, and all patients with ER‐positive tumours with moderate to poor NPI (>3.4) received adjuvant HT. ER‐negative patients classified into the poor prognostic NPI group were offered CMF. ER‐positive patients with positive lymph nodes were given HT coupled with CMF.

### METABRIC cohort (mRNA assessments)

Information on the METABRIC dataset is available via Curtis *et al* [26]. In brief, patient tumours were collected from five centres in the UK and Canada between 1977 and 2005. Median follow‐up was 141 months. Patients who were ER positive and/or lymph node negative did not receive adjuvant chemotherapy, whereas all ER‐negative and/or lymph node‐positive patients were administered adjuvant therapy. mRNA was isolated from primary patient tumours and assayed using the Illumina HT‐12 v 3 platform.

### Immunohistochemistry

Tissue microarrays (TMAs) have been described previously [27, 28]. Immunohistochemistry was performed using a Novolink Polymer Detection Kit (Leica, Newcastle, UK) according to the manufacturers' instructions, as described previously [29]. In brief, slides were deparaffinised in xylene and sequentially rehydrated in ethanol followed by water. Antigen retrieval was performed in 0.01 m sodium citrate buffer (pH 6.0), heated in a microwave for 10 min at 750 W followed by another 10 min at 450 W. Tissues were treated with Novolink Peroxidase Block, washed with TBS, followed by treatment with Novolink Protein Block. Tissues were treated for 1 h at room temperature with the primary antibodies, rabbit polyclonal anti‐CYP2S1 (Abcam Ab69650) and rabbit polyclonal anti‐CYP2W1 (Abcam Ab76666) diluted 1 in 100 and 1 in 50 respectively in Bond Primary Antibody Diluent (Leica). Tissues were washed with TBS prior to the application of Novolink Post Primary solution followed by incubation with NovoLink Polymer (anti‐mouse/rabbit IgG‐poly‐horseradish peroxidase). Immunohistochemical reactions were developed using 3,3′‐diaminobenzidine (DAB) solution (1:20 DAB chromogen in DAB substrate buffer) as the chromogenic substrate after which tissues were counterstained with haematoxylin. Tissues were dehydrated in alcohol and fixed in xylene, followed by mounting using DPX. Primary antibody specificity was confirmed by western blot on breast cancer cell lysates prior to use. Positive and negative controls were included with each run, with negative controls omitting primary antibody. CDK5 antibody, previously used by our group, was used as a positive control [30].

### IHC assessment and statistical analysis

Slides were scanned using a Nanozoomer Digital Pathology Scanner (Hamamatsu Photonics, Welwyn Garden City, UK) and staining assessed at ×200 total magnification. Cytoplasmic staining was assessed using a semi‐quantitative immunohistochemical H score (0–300), obtained from staining intensity, scored negative (0), weak (1), medium (2), or strong (3), multiplied by the percentage of area stained at that intensity [31]. Nuclear staining was assessed as the percentage of nuclei with any intensity of staining. CYP2S1 and CYP2W1 showed both cytoplasmic and nuclear staining. More than 30% of cores for each TMA were initially independently double assessed, with both assessors blinded to clinical outcome and each other's scores. Single measure intra‐class correlation coefficient was above 0.7 indicating good concordance between scorers. The primary assessor then scored the remaining cores. Data for mRNA and protein were stratified into high or low expression based on BCSS using X‐Tile software [32]. Statistical analysis was performed using SPSS (v26) and *p* < 0.05 was considered statistically significant. Spearman's correlation was used to establish correlation between CYP2S1 and CYP2W1 protein expression. Correlations between categorised protein/mRNA and clinicopathological variables were examined using Pearson's chi‐square of association (*χ* ^2^). Survival curves were plotted according to the Kaplan–Meier method with significance determined using the log‐rank test. For multivariate survival analysis, Cox proportional hazards regression model was used to test the statistical independence and significance of the predictors on overall survival.

## Results

### CYP2S1 and CYP2W1 protein expression

**Figure 1:**
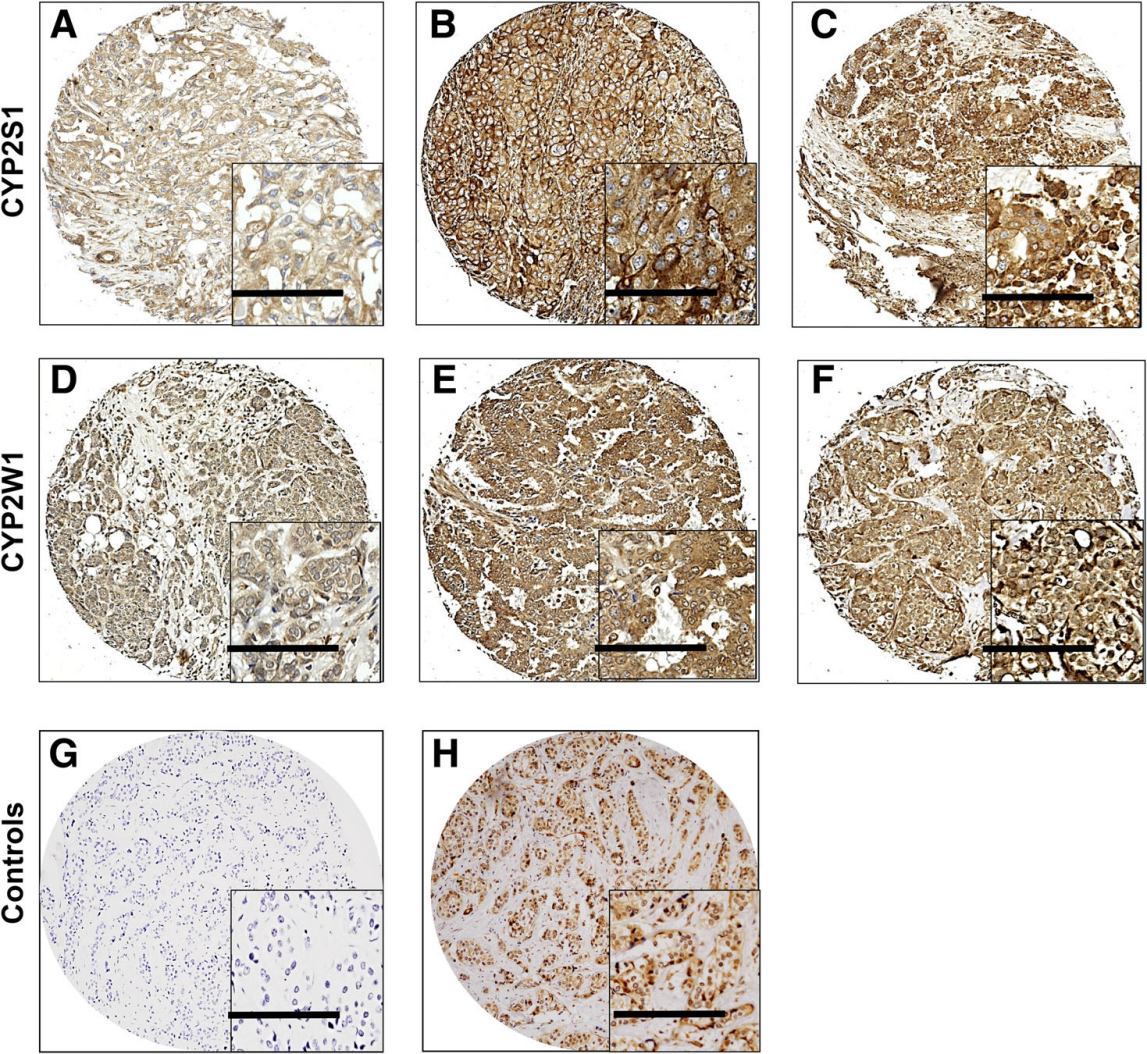
Representative photomicrographs of CYP2S1 and CYP2W1 staining. Photomicrographs of (A) low, (B) high, and (C) nuclear CYP2S1 staining. Photomicrographs of (D) low, (E) high, and (F) nuclear CYP2W1 staining. Representative negative control (G) and positive control (CDK5) (H). Images are shown at ×10 objective magnification with ×20 magnification inset panel. Scale bar represents 100 μm.

CYP2S1 and CYP2W1 expression was observed across all cores with staining intensity varying from weak to strong in both the cytoplasm and nucleus, and with heterogeneous expression observed between adjacent tumour cells. Representative staining patterns of low, high, and nuclear CYP2S1 and CYP2W1 are shown in Figure 1. A total of 1,216 and 1,193 patients were assessed for CYP2S1 and CYP2W1 expression respectively. Missing scores occurred due to core dropout or there being insufficient tumour cells to score i.e. a minimum of 20% tumour was required per core. Cytoplasmic CYP2S1 expression had a median *H*‐score of 150, with values ranging from 0 to 300, and an X‐tile generated cut‐point of 150, with 53.4% of cases (650/1,216) demonstrating low expression, and 46.6% of cases (566/1,216) with high expression. Nuclear expression had a median *H*‐score of 0 and ranged from 0 to 100, with an X‐tile cut‐point of 30, with 88.4% of cases (1,074/1,215) demonstrating low expression and 11.6% of cases (141/1,215) demonstrating high expression. Cytoplasmic CYP2W1 had a median *H*‐score of 130, ranging from 0 to 300, and an X‐tile cut‐point of 180, with 84.9% of cases (1,014/1,193) demonstrating low expression and 15.1% of cases (179/1,193) with high expression. Nuclear CYP2W1 had a median *H*‐score of 0 and ranged from 0 to 100, with an X‐tile cut‐point of 1, with 51.8% of cases (618/1,193) demonstrating low expression and 48.2% of cases (575/1,193) with high expression. Spearman's rank order correlation demonstrated significant, albeit weak, positive correlation of cytoplasmic CYP2S1 with cytoplasmic CYP2W1 (*r* ^2^ = 0.288, *p* < 0.001) and nuclear CYP2W1 (*r* ^2^ = 0.180, *p* < 0.001). Nuclear CYP2S1 was negatively correlated to cytoplasmic CYP2W1 (*r* ^2^ = −0.174, *p* < 0.001) and nuclear CYP2W1 (*r* ^2^ = −0.119, *p* < 0.001). Cytoplasmic CYP2S1 was correlated with its expression in the nucleus (*r* ^2^ = 0.288, *p* < 0.001). Cytoplasmic CYP2W1 was also correlated to nuclear CYP2W1 (*r* ^2^ = 0.326, *p* < 0.001).

### *CYP2S1* and *CYP2W1* mRNA expression

In the METABRIC cohort, *CYP2S1* mRNA expression ranged from 5.3 to 8.58 log_2_ intensity (median 6.02) and had an X‐tile generated cut‐point of 6.74, with 94.5% of cases (1,873/1,980) demonstrating low expression and 5.4% of cases (107/1,980) high expression. *CYP2W1* mRNA expression ranged from 4.81 to 6.29 log_2_ intensity (median 5.42), and had a cut‐point of 5.54, with 51.4% of cases (623/1,212) demonstrating low expression and 48.5% of cases (589/1,212) high expression. Spearman's correlation demonstrated that expression of *CYP2S1* mRNA was very weakly negatively correlated to *CYP2W1* mRNA (*r* ^2^ = −0.063, *p* = 0.005). This observation is similar to data obtained with nuclear CYP2S1 protein expression which was negatively correlated to both cytoplasmic and nuclear CYP2W1 protein.

### Association between CYP2S1 and CYP2W1 protein expression with clinicopathological variables

As shown in Table 1, low cytoplasmic CYP2S1 was significantly associated with ER‐positive status (*χ* ^2^ = 6.543, df = 1, *p =* 0.011), HER2‐negative disease (*χ* ^2^ = 10.721, df = 1, *p =* 0.001), ≥50 years (*χ* ^2^ = 11.496, df = 1, *p* < 0.001), grade 3 tumours (*χ* ^2^ = 24.785, df = 2, *p* < 0.001), intermediate NPI prognostic group (*χ* ^2^ = 12.138, df = 2, *p =* 0.002), high nuclear pleomorphism (*χ* ^2^ = 11.566, df = 2, *p =* 0.003), mitosis (*χ* ^2^ = 12.874, df = 2, *p <* 0.001), and ductal carcinomas (*χ* ^2^ = 14.790, df = 6, *p =* 0.022). Low nuclear CYP2S1 was associated with HER2‐negative disease (*χ* ^2^ = 6.396, df = 1, *p =* 0.011), grade 3 tumours (*χ* ^2^ = 9.422, df = 2, *p =* 0.009), intermediate NPI (*χ* ^2^ = 7.415, df = 2, *p =* 0.025), mitosis (*χ* ^2^ = 12.492, df = 2, *p =* 0.002), and ductal carcinomas (*χ* ^2^ = 17.161, df = 6, *p =* 0.007).

**Table 1:** Clinicopathological associations with cytoplasmic and nuclear CYP2S1 protein expression.

	CYP2S1					
	Cytoplasmic expression			Nuclear expression		
Clinicopathological parameters	Low	High	*P* value	Low	High	*P* value
Age						
<50 years	198 (14.9%)	220 (16.5%)	**<0.001**	366 (27.5%)	816 (61.4%)	0.278
≥50 years	523 (39.3%)	389 (29.2%)		52 (3.9%)	95 (7.1%)	
Tumour size						
<20 mm	436 (32.8%)	370 (27.9)	0.966	714 (53.8%)	92 (6.9%)	0.627
≥20 mm	283 (21.3%)	239 (18.0%)		466 (35.1%)	57 (4.1%)	
Nodal stage						
1	444 (33.9%)	375 (28.3%)	0.919	738 (55.7%)	86 (6.5%)	0.592
2	197 (14.9%)	173 (13%)		324 (24.5%)	46 (3.5%)	
3	72 (5.4%)	60 (4.5%)		116 (8.8%)	16 (1.2%)	
Tumour grade						
1	110 (8.3%)	87 (6.5%)	**<0.001**	183 (13.8%)	15 (1.1%)	**0.009**
2	333 (25.1%)	208 (15.7%)		490 (36.9%)	51 (3.8%)	
3	277 (20.8%)	314 (23.6%)		508 (38.3%)	65 (6.2%)	
ER status						
Negative	122 (9.2%)	137 (10.3%)	**0.011**	224 (16.9%)	35 (2.6%)	0.161
Positive	599 (45%)	472 (35.5%)		957 (72.1%)	112 (8.4%)	
PgR status						
Negative	279 (22.4%)	244 (19.6%)	0.947	459 (36.9%)	64 (5.1%)	0.278
Positive	386 (31%)	335 (26.5%)		646 (52%)	74 (6%)	
NPI category						
Good (≤3.4)	265 (20.0%)	172 (13.0%)	**0.002**	403 (30.4%)	34 (2.6%)	**0.025**
Intermediate (3.41–5.4)	340 (25.7%)	340 (25.7%)		592 (44.7%)	88 (6.6%)	
Poor (>5.4)	112 (8.5%)	96 (7.2%)		182 (13.7%)	25 (1.9%)	
Tubule formation						
1	47 (3.6%)	45 (3.4%)	0.809	85 (6.5%)	7 (0.5%)	0.200
2	209 (15.9%)	172 (13.1%)		344 (26.2%)	42 (2.7%)	
3	456 (34.7%)	387 (29.4%)		740 (56.3%)	93 (7.8%)	
Pleomorphism						
1	11 (0.8%)	10 (0.8%)	**0.003**	19 (1.4%)	2 (0.2%)	0.562
2	234 (17.8%)	147 (11.2%)		344 (26.2%)	37 (2.8%)	
3	467 (35.5%)	447 (34%)		806 (61.3%)	107 (8.1%)	
Mitosis						
1	393 (29.9%)	248 (18.9%)	**<0.001**	582 (44.3%)	59 (4.5%)	**0.002**
2	124 (9.4%)	128 (9.7%)		231 (17.6%)	21 (1.6%)	
3	194 (14.8%)	227 (17.3%)		355 (27%)	65 (5%)	
HER2 status						
Negative	628 (50.6%)	510 (41.1%)	**0.001**	1,021(82.3%)	116 (9.3%)	**0.011**
Positive	40 (3.2%)	64 (5.2%)		85 (6.8%)	19 (1.5%)	
Triple negative disease						
Negative	616 (47%)	499 (38%)	0.091	994 (75.8%)	120 (9.2%)	0.869
Positive	96 (7.3%)	101 (7.7%)		175 (13.3%)	22 (1.7%)	
Vascular invasion						
Negative	500 (37.7%)	430 (32.4%)	0.701	353 (26.6%)	44 (3.3%)	0.998
Positive	218 (16.4%)	179 (13.5%)		826 (62.3%)	103 (7.8%)	
Tumour type						
Ductal (including mixed)	608 (45.7%)	537 (40.4%)	**0.022**	1,030 (77.6%)	114 (8.6%)	**0.007**
Lobular	70 (5.3%)	38 (2.9%)		85 (6.4%)	23 (1.7%)	
Medullary‐like	1 (0.1%)	8 (0.6%)		6 (0.5%)	3 (0.2%)	
Miscellaneous	5 (0.4%)	5 (0.4%)		9 (0.7%)	1 (0.1%)	
Mixed NST and lobular	1 (0.1%)	0 (0.0%)		1 (0.1%)	0 (0.0%)	
Special type	34 (2.6%)	20 (1.5%)		48 (3.6%)	6 (0.5%)	
Tubular	1 (0.1%)	1 (0.1%)		2 (0.2%)	0 (0.0%)	

The *P* values are derived using the Pearson *χ* ^2^ test of association. Significant *P* values (<0.05) are indicated in bold. The number and percentage of observations for cohort are shown for each clinicopathological variable.

Low cytoplasmic CYP2W1 protein expression (Table 2) was significantly associated with patients ≥50 years (*χ* ^2^ = 9.325, df = 1, *p =* 0.002), HER2‐negative disease (*χ* ^2^ = 8.903, df = 1, *p =* 0.003), intermediate NPI (*χ* ^2^ = 8.678, df = 2, *p =* 0.013), and mitosis (*χ* ^2^ = 9.516, df = 2, *p =* 0.009). Nuclear expression did not demonstrate any association with clinicopathological parameters.

**Table 2:** Clinicopathological associations with cytoplasmic and nuclear CYP2W1 protein expression.

	CYP2W1					
	Cytoplasmic expression			Nuclear expression		
Clinicopathological parameters	Low	High	*P* value	Low	High	*P* value
Age						
<50 years	335 (25.6%)	81 (6.2%)	**0.002**	206 (15.7%)	210 (16%)	0.390
≥50 years	777 (59.4%)	116 (8.9%)		465 (35.5%)	428 (32.7%)	
Tumour size						
<20 mm	665 (50.9%)	130 (9.9%)	0.107	404 (30.9%)	391 (29.9%)	0.740
≥20 mm	445 (34%)	67 (5.1%)		265 (20.3%)	247 (18.9%)	
Nodal stage						
1	686 (52.6%)	127 (9.7%)	0.218	422 (32.3%)	391 (39%)	0.796
2	303 (23.3%)	57 (4.4%)		181 (13.9%)	180 (13.8%)	
3	118 (9.0%)	13 (1%)		65 (5.0%)	66 (5.1%)	
Tumour grade						
1	161 (12.3%)	26 (2%)	0.185	82 (6.3%)	105 (8%)	0.086
2	464 (35.5%)	71 (5.4%)		284 (21.7%)	261 (19.2%)	
3	468 (37.2%)	100 (7.6%)		304 (23.2%)	282 (21.6%)	
ER status						
Negative	209 (16%)	47 (3.6%)	0.107	137 (10.5%)	119 (9.1%)	0.405
Positive	902 (68.9%)	151 (11.5%)		533 (40.7%)	520 (39.7%)	
PgR status						
Negative	442 (36.3%)	70 (5.8%)	0.256	254 (20.9%)	258 (21.2%)	0.322
Positive	592 (48.6%)	113 (9.6%)		370 (30.4%)	335 (27.5%)	
NPI category						
Good (≤3.4)	373 (28.6%)	57 (4.4%)	**0.013**	211 (16.2%)	219 (16.8%)	0.518
Intermediate (3.41–5.4)	550 (42.2%)	119 (9.1%)		352 (27.0%)	317 (24.3%)	
Poor (>5.4)	184 (14.1%)	21 (1.6%)		105 (8.1%)	100 (7.7%)	
Tubule formation						
1	75 (5.8%)	15 (1.2%)	0.624	36 (2.8%)	54 (4.2%)	0.081
2	303 (23.4%)	59 (4.6%)		185 (14.3%)	177 (13.7%)	
3	722 (55.8%)	121 (9.3%)		442 (34.1%)	401 (31%)	
Pleomorphism						
1	15 (1.2%)	1 (0.1%)	0.050	5 (0.4%)	11 (0.8%)	0.275
2	332 (25.6%)	44 (3.4%)		193 (14.9%)	183.5 (14.1%)	
3	752 (58.1%)	150 (11.6%)		465 (35.9%)	438 (33.8%)	
Mitosis						
1	553 (42.8%)	76 (5.9%)	**0.009**	314 (24.3%)	315 (24.4%)	0.503
2	207 (16%)	40 (3.1%)		124 (9.6%)	123 (99.5%)	
3	338 (26.1%)	79 (6.1%)		223 (17.2%)	194 (15.0%)	
HER2 status						
Negative	964 (78.4%)	164 (13.3%)	**0.003**	579 (47.1%)	549 (44.7%)	0.464
Positive	75 (6.1%)	26 (2.1%)		48 (3.9%)	49.5 (4.3%)	
Triple negative disease						
Negative	937 (72.5%)	158 (12.2%)	0.164	550 (42.6%)	545 (42.2%)	0.187
Positive	161 (12.5%)	36 (2.8%)		109 (8.4%)	88 (6.9%)	
Vascular invasion						
Negative	772 (59.1%)	147 (11.3%)	0.156	455 (34.8%)	464 (35.5%)	0.068
Positive	337 (25.8%)	50 (3.8%)		213 (16.3%)	174 (13.3%)	
Tumour type						
Ductal (including mixed)	954 (72.9%)	177 (13.5%)	0.050	587 (44.9%)	544 (41.6%)	0.050
Lobular	100 (7.6%)	6 (0.5%)		53 (4.1%)	53 (4.1%)	
Medullary‐like	8 (0.6%)	3 (0.2%)		5 (0.4%)	6 (0.5%)	
Miscellaneous	9 (0.7%)	1 (0.1%)		4 (0.3%)	6 (0.5%)	
Special type	37 (2.8%)	10 (0.8%)		18 (1.4%)	29 (2.2%)	
Tubular	3 (0.2%)	0 (0.0%)		3 (0.2%)	0 (0.0%)	

The *P* values are derived using the Pearson *χ* ^2^ test of association. Significant *P* values are indicated in bold. The number and percentage of observations for cohort are shown for each clinicopathological variable.

From the METABRIC cohort (supplementary material, Table S1), low *CYP2S1* mRNA expression was significantly associated with positive progesterone receptor (PgR) (*χ* ^2^ = 10.400, df = 1, *p* = 0.001), HER2‐negative status (*χ* ^2^ = 11.652, df = 1, *p* = 0.001), and luminal A cancers (*χ* ^2^ = 15.377, df = 5, *p* = 0.009). The expression of low *CYP2W1* mRNA was significantly associated with tumour size ≥20 mm (*χ* ^2^ = 6.919, df = 1, *p* = 0.009) and luminal A cancers (*χ* ^2^ = 13.431, df = 5, *p* = 0.020). The association of low *CYP2S1* mRNA with HER2‐negative status, and low *CYP2S1/CYP2W1* with luminal A tumours agrees with results obtained in the Nottingham cohort protein assessments.

### Associations between CYP2S1 and CYP2W1 protein expression with survival

**Figure 2:**
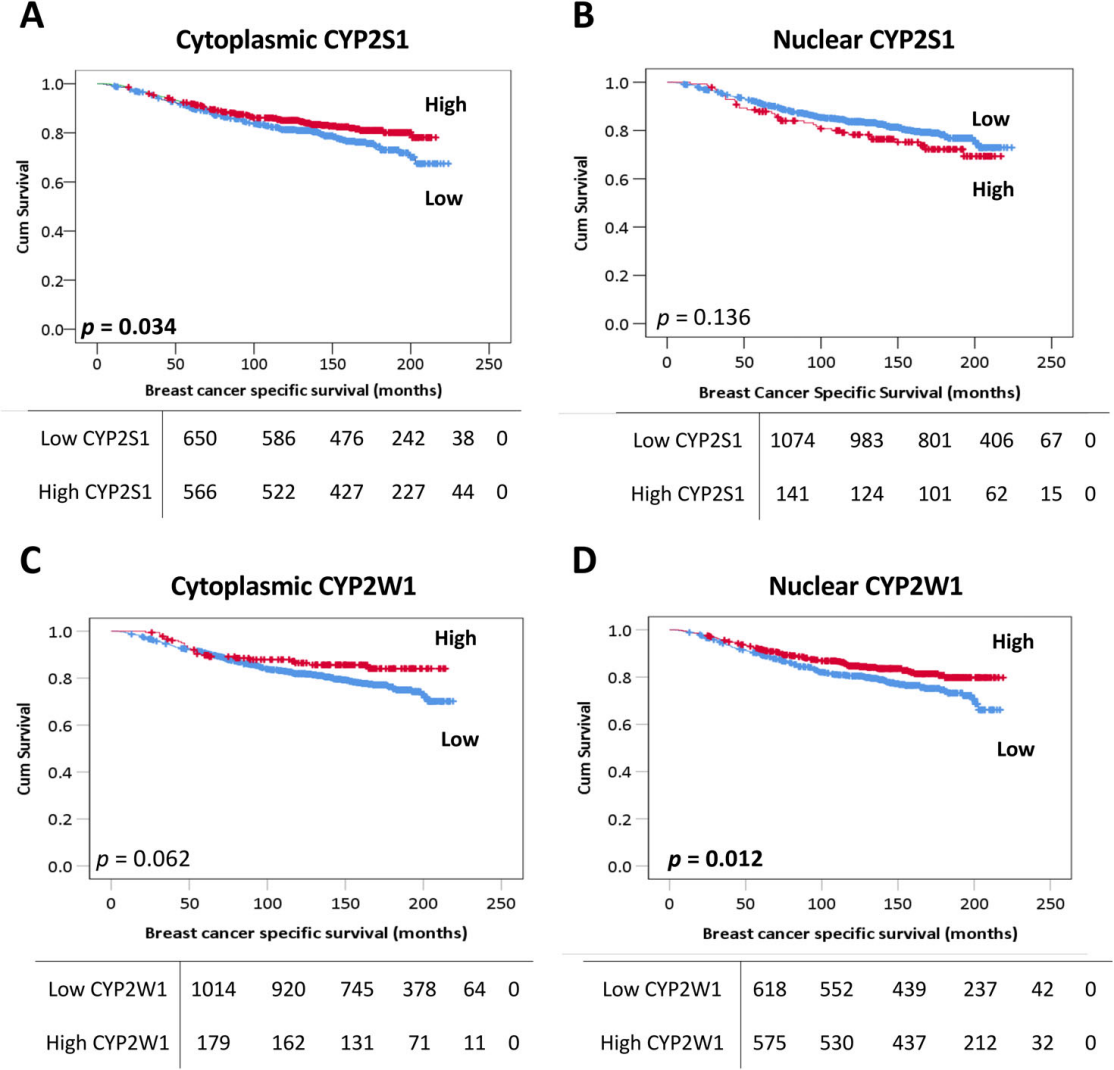
Kaplan–Meier survival analysis of CYP2S1 and CYP2W1 and breast cancer specific survival. Survival curves showing the impact of low (blue line) and high (red line) (A) cytoplasmic CYP2S1, (B) nuclear CYP2S1 expression, (C) cytoplasmic CYP2W1, and (D) nuclear CYP2W1 expression. Significance was determined using the log‐rank test. The numbers shown below the Kaplan–Meier survival curves are the number of patients at risk at the specified month. Significant *P* values (<0.05) are indicated in bold.

Low cytoplasmic, but not nuclear, expression of CYP2S1 was significantly associated with poor breast cancer specific survival (*p* = 0.034) in the total patient cohort (Figure 2A,B). Low nuclear, but not cytoplasmic, expression of CYP2W1 was also significantly associated with adverse breast cancer specific survival (*p =* 0.012) (Figure 2D and 2C respectively). When potentially confounding factors were included in multivariate assessment (tumour size, grade, nodal stage, vascular invasion status, ER, PgR, and HER2 status), low cytoplasmic expression of CYP2S1 was significantly independently associated with adverse survival (hazard ratio [HR]: 0.639; 95% confidence interval [CI]: 0.483–0.846; *p* = 0.002) (Table 3). Similarly, multivariate analysis of nuclear CYP2W1 expression remained independently significantly associated with poor survival (HR: 0.677; 95% CI: 0.510–0.898; *p* = 0.007) (Table 4). Unlike protein, mRNA expression of both *CYP2S1* (*p* = 0.279) and *CYP2W1* (*p* = 0.121) was not significantly associated with survival in the METABRIC cohort (supplementary material, Figure S1).

**Table 3:** Multivariate Cox proportional hazards analysis for predictors of overall survival for cytoplasmic CYP2S1 expression.

			95% CI for Exp(*B*)	
Variables	*P* value	Exp(*B*)	Lower	Upper
CYP2S1 (cytoplasmic)	**0.002**	0.639	0.483	0.846
Tumour size	0.069	0.766	0.575	1.021
Tumour grade	**<0.001**	1.852	1.411	2.432
Nodal stage	**<0.001**	1.783	1.467	2.167
ER status	0.810	0.955	0.654	1.394
PgR status	**<0.001**	1.857	1.329	2.593
HER2 status	**0.037**	0.669	0.458	0.976
Vascular invasion	**0.003**	1.594	1.176	2.161

Exp(*B*) is used to denote hazard ratio. Significant *P* values (<0.05) are indicated in bold.

**Table 4:** Multivariate Cox proportional hazards analysis for predictors of overall survival for nuclear CYP2W1 expression.

			95% CI for Exp(*B*)	
Variables	*P* value	Exp(*B*)	Lower	Upper
CYP2W1 (nuclear)	**0.007**	0.677	0.510	0.898
Tumour size	0.159	0.806	0.598	1.088
Tumour grade	**<0.001**	1.770	1.347	2.326
Nodal stage	**<0.001**	1.852	1.518	2.259
ER status	0.878	0.970	0.660	1.425
PgR status	**<0.001**	2.002	1.425	2.813
HER2 status	0.083	0.710	0.483	1.046
Vascular invasion	**0.001**	1.686	1.235	2.302

Exp(*B*) is used to denote hazard ratio. Significant *P* values (<0.05) are indicated in bold.

### Association between CYP2S1 and CYP2W1 protein expression with survival in ER‐positive and ER‐negative disease

**Figure 3:**
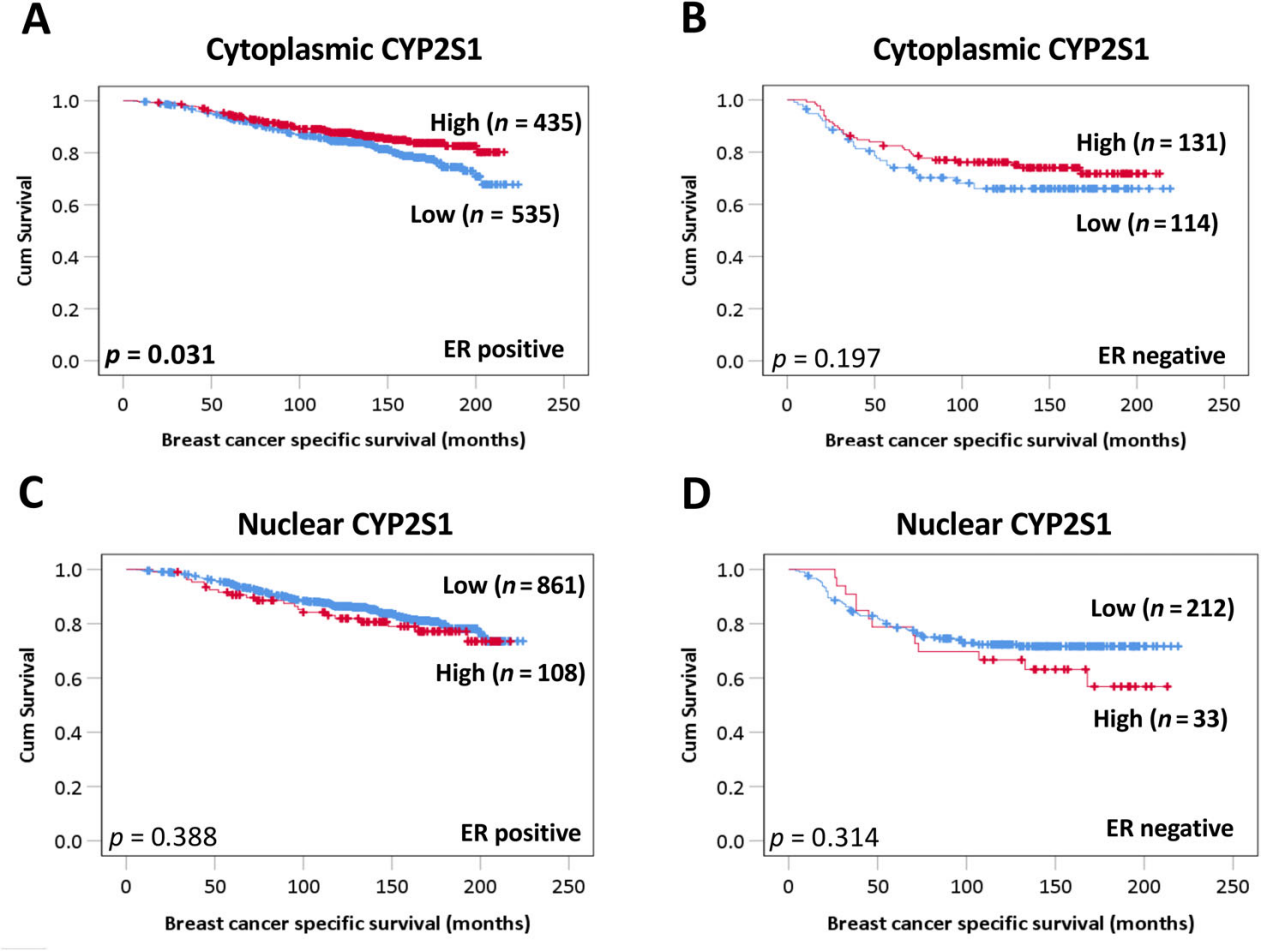
Kaplan–Meier analysis of CYP2S1 and survival in ER‐positive and ER‐negative breast cancers. Survival curves showing the impact of low (blue line) and high (red line) CYP2S1 expression in ER‐positive and ER‐negative patients. Cytoplasmic CYP2S1 expression in (A) ER‐positive and (B) ER‐negative patients. Nuclear CYP2S1 in (C) ER‐positive and (D) ER‐negative patients. Significance was determined using the log‐rank test. Significant *P* values (<0.05) are indicated in bold.

The involvement of CYP2W1 in the metabolism and oxidation of estrogen [33], along with the strong association of cytoplasmic CYP2S1 and nuclear CYP2W1 protein with ER‐positive cancers (Tables 1 and 2), suggests that these enzymes may be important as prognostic markers in ER‐positive disease. As shown in Figure 3, low cytoplasmic CYP2S1 was significantly associated with adverse breast cancer specific survival in ER‐positive patients (*p* = 0.031), but not in ER‐negative disease (*p* = 0.197). Multivariate analysis of cytoplasmic CYP2S1 expression in ER‐positive cancers remained independently significantly associated with poor survival (HR: 0.578; 95% CI: 0.419–0.796; *p* = 0.001) (Table 5). There was no significant association between nuclear CYP2S1 and survival in ER‐positive (*p* = 0.388) or ER‐negative cancers (*p* = 0.314).

**Table 5:** Multivariate Cox proportional hazards analysis for predictors of overall survival for cytoplasmic CYP2S1 expression in ER‐positive cancers.

			95% CI for Exp(*B*)	
Variables	*P* value	Exp(*B*)	Lower	Upper
CYP2S1 (cytoplasmic)	**0.001**	0.578	0.419	0.796
Tumour size	**0.006**	1.583	1.144	2.190
Tumour grade	**<0.001**	2.064	1.565	2.722
Nodal stage	**<0.001**	1.839	1.475	2.292
PgR status	**0.001**	0.570	0.414	0.785
HER2 status	0.455	0.834	0.518	1.343
Vascular invasion	0.086	1.347	0.959	1.893

Exp(*B*) is used to denote hazard ratio. Significant *P* values (<0.05) are indicated in bold.

**Figure 4:**
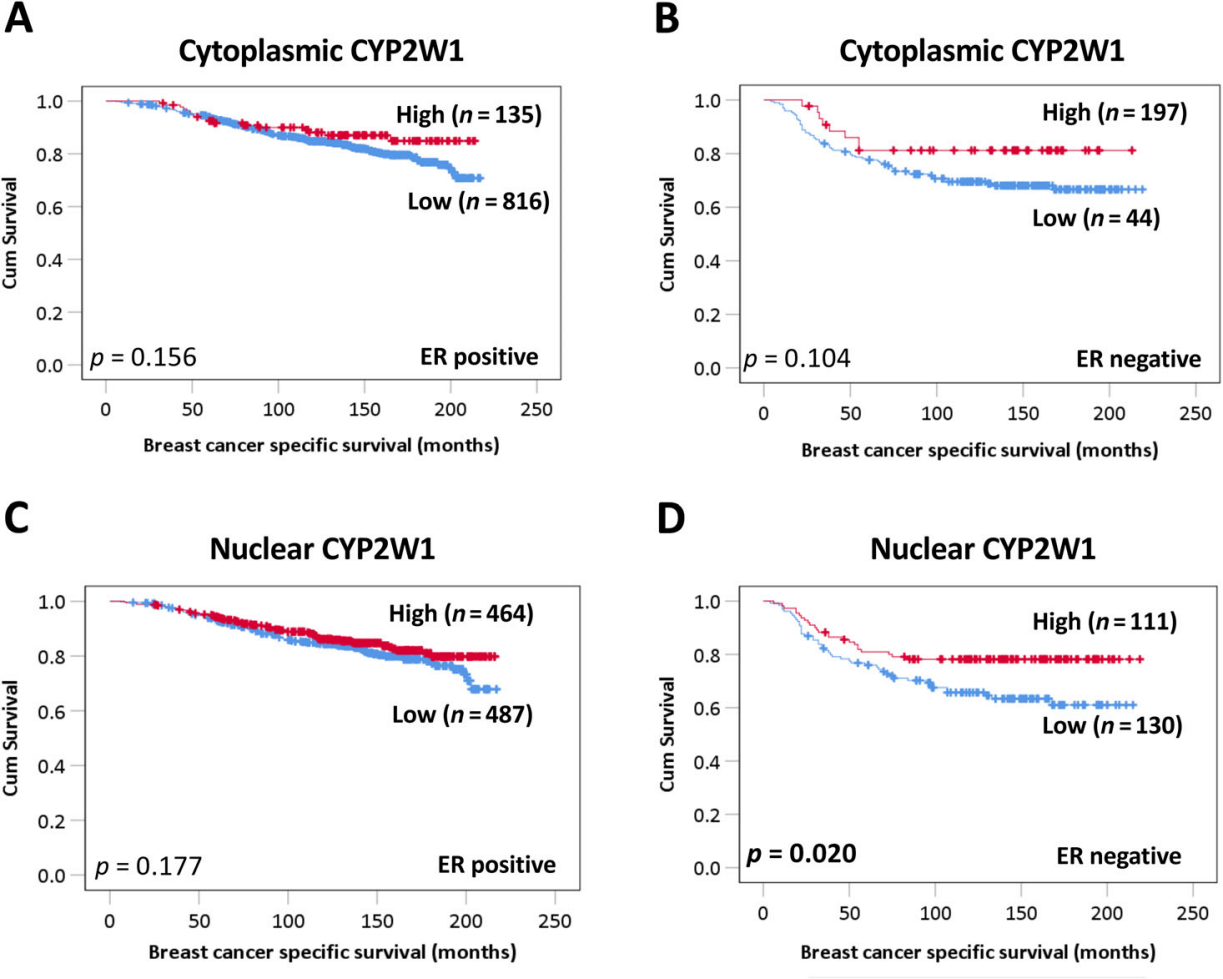
Kaplan–Meier analysis of CYP2W1 and survival in ER‐positive and ER‐negative breast cancers. Survival curves showing the impact of low (blue line) and high (red line) CYP2W1 expression in ER‐positive and ER‐negative patients. Cytoplasmic CYP2W1 expression in (A) ER‐positive and (B) ER‐negative patients. Nuclear CYP2W1 in (C) ER‐positive and (D) ER‐negative patients. Significance was determined using the log‐rank test. Significant *P* values (<0.05) are indicated in bold.

As shown in Figure 4, low nuclear CYP2W1 protein expression was also significantly associated with poor survival in ER‐negative patients (*p* = 0.020), but not in ER‐positive disease (*p* = 0.177). The expression of cytoplasmic CYP2W1 was not associated with survival in either ER‐positive (*p* = 0.156) or ER‐negative patients (*p* = 0.104).

### Associations of combined cytoplasmic and nuclear CYP expression with survival

**Figure 5:**
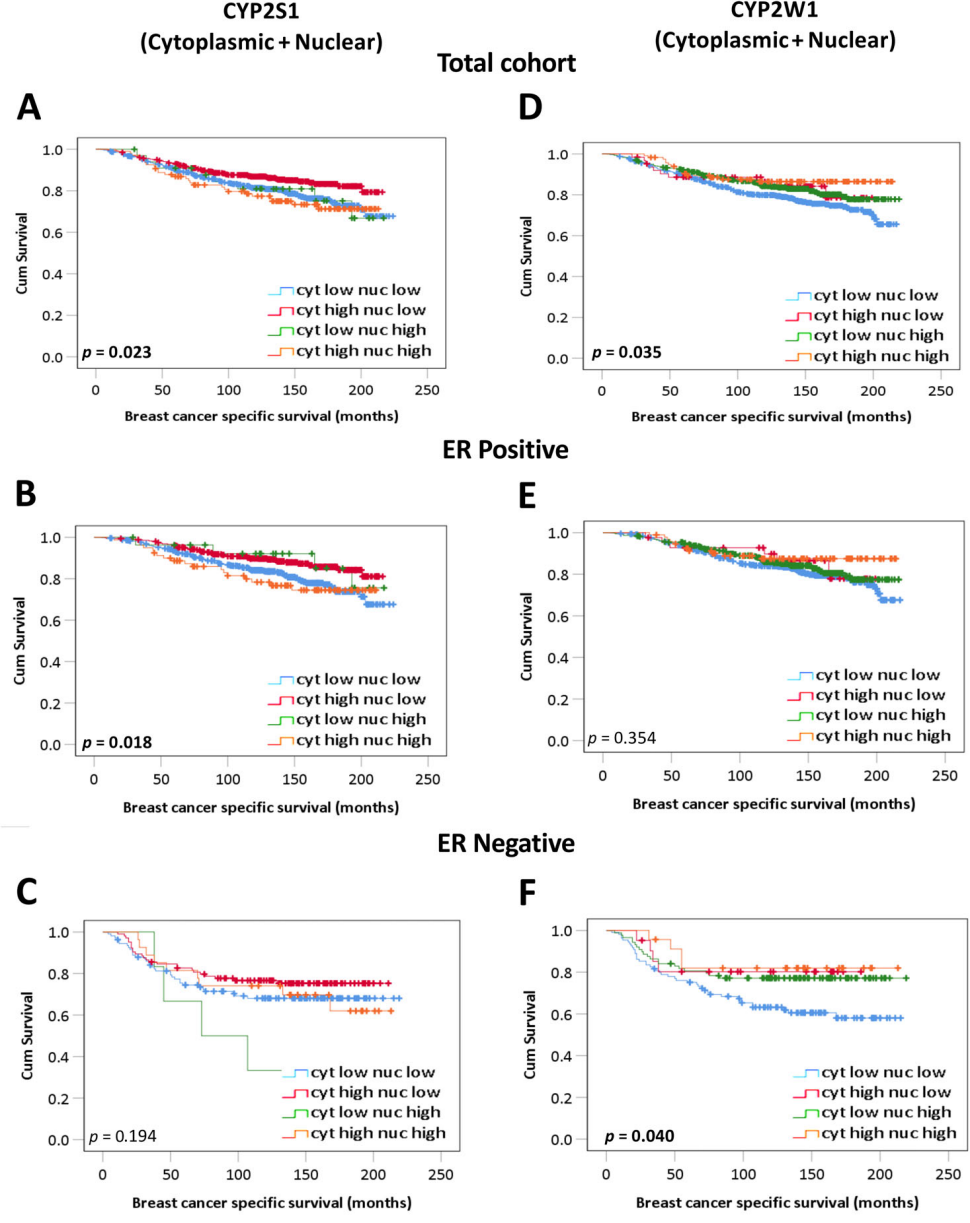
Kaplan–Meier analysis of combined cytoplasmic and nuclear CYP2S1 or CYP2W1 on breast cancer specific survival. The effect of combined cytoplasmic and nuclear CYP2S1 in (A) the total patient cohort, (B) ER‐positive, and (C) ER‐negative patients. The effect of combined cytoplasmic and nuclear CYP2W1 in (D) the total patient cohort, (E) ER‐positive, and (F) ER‐negative patients. Survival curves showing the impact of low cytoplasmic and low nuclear (blue line); high cytoplasmic and low nuclear (red line); low cytoplasmic and high nuclear (green line); and high cytoplasmic and high nuclear (orange line) expression. Significance was determined using the log‐rank test. Significant *P* values (<0.05) are indicated in bold.

Combined expression of cytoplasmic and nuclear CYP2S1 or CYP2W1 was assessed to determine association with survival in the total patient cohort, ER‐positive and ER‐negative cancers. Data were stratified into the four groups – low cytoplasmic and low nuclear, high cytoplasmic and low nuclear, low cytoplasmic and high nuclear, and high cytoplasmic and high nuclear expression. High expression of combined cytoplasmic and nuclear CYP2S1 was significantly associated with adverse survival in the total cohort (*p* = 0.023, Figure 5A) and in ER‐positive patients (*p* = 0.018, Figure 5B). Low expression of combined cytoplasmic and nuclear CYP2W1 was associated with adverse survival in the total patient cohort (*p* = 0.035, Figure 5D) and in the ER‐negative subgroup (*p* = 0.040, Figure 5F). Multivariate analysis of combined cytoplasmic and nuclear CYP2W1 expression remained independently significantly associated with poor survival in the total patient cohort (HR: 1.228; 95% CI: 1.088–1.387; *p =* 0.001), and in ER‐negative cancers (HR: 1.211; 95% CI: 1.047–1.400; *p =* 0.010), as shown in Tables 6 and 7 respectively. None of the combined groupings of cytoplasmic and nuclear CYP2S1 or CYP2W1 showed any significant associations in ER‐negative (*p* = 0.194, Figure 5C) or ER‐positive breast cancers (*p* = 0.354, Figure 5E) respectively.

**Table 6:** Multivariate Cox proportional hazards analysis for predictors of overall survival for cytoplasmic and nuclear CYP2W1 expression in the total patient cohort.

			95% CI for Exp(*B*)	
Variables	*P* value	Exp(*B*)	Lower	Upper
CYP2W1 (cytoplasmic and nuclear)	**<0.001**	1.228	1.088	1.387
Tumour size	**0.021**	1.381	1.049	1.818
Tumour grade	**<0.001**	1.599	1.246	2.052
Nodal stage	**<0.001**	1.875	1.558	2.257
ER status	0.522	0.891	0.625	1.269
PgR status	**<0.001**	0.567	0.415	0.776
HER2 status	0.514	1.120	0.796	1.577
Vascular invasion	**<0.001**	1.803	1.358	2.393

Exp(*B*) is used to denote hazard ratio. Significant *P* values (<0.05) are indicated in bold.

**Table 7:** Multivariate Cox proportional hazards analysis for predictors of overall survival for cytoplasmic and nuclear CYP2W1 expression in ER‐negative cancers.

			95% CI for Exp(*B*)	
Variables	*P* value	Exp(*B*)	Lower	Upper
CYP2W1 (cytoplasmic and nuclear)	**0.010**	1.211	1.047	1.400
Tumour size	**0.028**	1.452	1.042	2.024
Tumour grade	**<0.001**	1.904	1.447	2.505
Nodal stage	**<0.001**	1.897	1.521	2.366
PgR status	**<0.001**	0.548	0.398	0.755
HER2 status	0.291	0.771	0.476	1.249
Vascular invasion	0.110	1.323	0.939	1.864

Exp(*B*) is used to denote hazard ratio. Significant *P* values (<0.05) are indicated in bold.

### Association of combined CYP2S1 and CYP2W1 expression with survival

**Figure 6:**
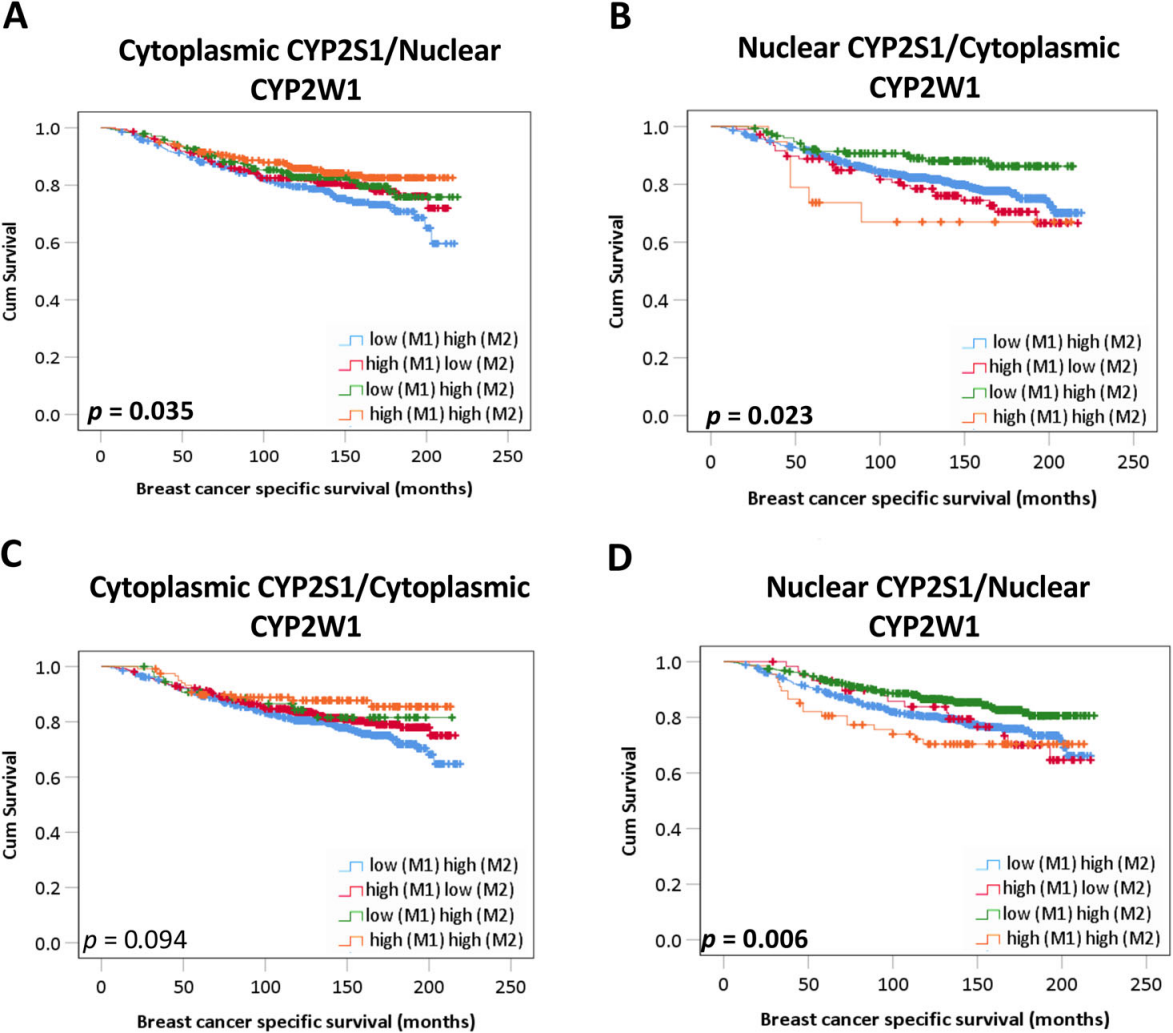
Kaplan–Meier analysis of combined CYP2S1 and CYP2W1 on breast cancer specific survival. Survival curves showing the impact of (A) cytoplasmic CYP2S1 and nuclear CYP2W1, (B) nuclear CYP2S1 and cytoplasmic CYP2W1, (C) cytoplasmic CYP2S1 and CYP2W1, and (D) nuclear CYP2S1 and CYP2W1 in the total patient cohort. Low–low expression of both markers (1 and 2) is depicted as blue line; high (marker 1) and low (marker 2) as red line; low (marker 1) and high (marker 2) as green line, and high (marker 1) and high (marker 2) as orange line. Significance was determined using the log‐rank test. Significant *P* values (<0.05) are indicated in bold.

In addition to looking at single marker expression, combined expression of both CYPs (CYP2S1 and CYP2W1) was also examined to assess the effects of marker combinations on breast cancer specific survival. Low cytoplasmic CYP2S1 and nuclear CYP2W1 expression (Figure 6A) (*p* = 0.035), and high nuclear CYP2S1 and cytoplasmic CYP2W1 expression (*p* = 0.023) (Figure 6B), were significantly associated with adverse breast cancer specific survival in the total patient cohort. High expression of nuclear CYP2S1 and of CYP2W1 (*p* = 0.006) (Figure 6D) was also significantly associated with adverse breast cancer specific survival (*p* = 0.006). Multivariate analysis of high nuclear CYP2S1 and CYP2W1 remained independently significantly associated with poor survival (HR: 1.225; 95% CI: 1.082–1.388; *p =* 0.001) (Table 8), in the total patient cohort. Combined cytoplasmic CYP2S1 and CYP2W1 expression (Figure 6C) was not significantly associated with survival (*p* = 0.094).

**Table 8:** Multivariate Cox proportional hazards analysis for predictors of overall survival for combined nuclear CYP2S1 and CYP2W1 expression.

			95% CI for Exp(*B*)	
Variables	*P* value	Exp(*B*)	Lower	Upper
CYP2S1 and CYP2W1 (nuclear)	**0.001**	1.225	1.082	1.388
Tumour size	**0.010**	1.446	1.090	1.919
Tumour grade	**0.001**	1.524	1.178	1.970
Nodal stage	**<0.001**	1.893	1.562	2.295
ER status	0.593	0.906	0.629	1.303
PgR status	**<0.001**	0.552	0.400	0.762
HER2 status	0.631	1.090	0.768	1.546
Vascular invasion	**<0.001**	1.708	1.273	2.292

Exp(*B*) is used to denote hazard ratio. Significant *P* values (<0.05) are indicated in bold.

## Discussion

This study assessed CYP2S1 and CYP2W1 protein expression in a large independent cohort of early‐stage invasive breast cancer patients (Nottingham cohort) to evaluate associations with various clinicopathological and survival parameters. The mRNA expression of both CYPs was also assessed in the METABRIC cohort.

CYP2S1 and CYP2W1 belong to the CYP2 family of cytochrome P450 enzymes and share 40% sequence homology [3, 34, 35]. They are expressed in epithelial cells [33, 35, 36], and upregulated, *in vitro*, in breast cancer cells treated with anti‐cancer agents such as 5F‐203 and GW‐610 [37]. Both these proteins are reportedly upregulated in hypoxia by HIF‐1α [38, 39], and involved in the bioreductive activation of the HAP, AQ4N [24]. Current data show that cytoplasmic CYP2S1 and nuclear CYP2W1 protein expression were specific to ER‐positive cancers. *CYP2S1* and *CYP2W1* mRNAs were also associated with luminal A cancers in the METABRIC cohort, all of which are consistent with the reported role of CYP enzymes in the metabolism and oxidation of estrogens [33]. Although a role for CYP2W1 in the metabolism of β‐estradiol has been previously reported [33], there are no studies linking CYP2S1 to estrogen metabolism. Further studies are required to evaluate the role of CYP2S1 and CYP2W1 in estrogen metabolism and ER‐positive cancers. Low expression of CYP2S1 and CYP2W1 identified a subgroup of ER‐positive, HER2‐negative patients, above 50 years of age, characterised by high grade, pleomorphism, mitosis, and intermediate NPI category. Clinically, ER‐positive patients who fall into the intermediate‐risk NPI category can be recommended additional cytotoxic chemotherapy in addition to HR therapy to reduce the risk of recurrence [40]. Such data suggest that low expression of cytoplasmic CYP2S1 or CYP2W1 protein identifies a subgroup of ER‐positive patients with higher proliferative and metastatic potential who may be eligible for additional, more targeted, treatments [41, 42]. In the case of advanced ER‐positive disease, targeted treatments include a combination of aromatase inhibitor with the CDK 4/6 inhibitors palbociclib/abemaciclib [43]. Bisphosphonates (zoledronic acid or sodium clodronate) are also prescribed for post‐menopausal women with a high risk of recurrence [44]. Other targeted treatments include the PARP inhibitor, olaparib which is approved for use in patients with *BRCA* mutation [45, 46]. For metastatic HER2 enriched breast cancer patients, trastuzumab‐DM1 is administered as second line therapy in patients who progress after the initial trastuzumab‐taxane therapy [47].

Furthermore, this subgroup of patients identified by low CYP2S1 and CYP2W1 could potentially become candidates for treatment with agents that are reliant on CYP2S1 and CYP2W1 for metabolism, with a number currently under evaluation. Hlaváč *et al* reported that patients with CYP2W1 expression responded better to a NACT regimen based on 5‐fluorouracil, adriamycin, cyclophosphamide or 5 fluorouracil, epirubicin, cyclophosphamide, and taxanes, which suggest that these commonly used breast cancer chemotherapy agents may be metabolised by CYP2W1 [13]. CYP2S1 and CYP2W1 are also involved in the metabolism of 5F‐203 and GW‐610 in breast and colorectal cancer cells [37].

The distinction between benign and malignant tumours is dependent on various factors, such as increased proliferation, invasiveness, and metastatic potential [48]. In breast cancer, assessment of tumour cell proliferation is one of the most integral parts of breast cancer grading [49]. Evaluation of proliferation involves assessing mitotic activity by counting the number of cells present in the M phase of the cell cycle [50]. Higher mitotic figures increase the probability of developing distant metastasis and shorter survival [51]. In the current study, CYP2S1 and CYP2W1 show association with increased mitosis, suggesting that the current CYP enzymes may be involved in cell proliferation and division. Studies have reported elevated levels of CYP2S1 in psoriasis, a condition that is mainly characterised by hyper‐proliferation of keratinocytes [52]. CYP2S1 has also been reported to modulate expression of prostaglandin E2 (PGE_2_) to stimulate cellular proliferation, and migration in the human bronchial epithelial BEAS‐2B cell line [53]. Furthermore, increased expression of PGE_2_ (regulated by CYP2S1) was found to increase cellular proliferation in colorectal cancer cells [54]. CYP2W1 has also been reported to be expressed in rapidly proliferating tissues [33], with such an association potentially being through retinoid which is a well‐known substrate of CYP2W1 [55]. Retinoid has anti‐cancer properties and functions to arrest cellular proliferation and induce cell differentiation [56]. The presence of CYP2W1 in breast cancers inhibits metabolism of retinoid into retinoic acid, reducing its anti‐proliferative effect in tumours [57]. Furthermore, inhibition of CYP1A1 in the luminal MCF‐7 and triple negative MDAMB‐231 breast cancer cell lines decreased proliferation and clonogenic survival [58], all of which suggests an association of CYP proteins with cellular proliferation, although further studies are required to confirm this.

In addition to estrogen, CYPs also metabolise various anti‐cancer drugs used in breast cancer [59, 60]. Tamoxifen, used in the treatment of ER/PgR‐positive cancers, is reduced by the CYP enzymes into its active metabolites N‐desmethyl‐tamoxifen, 4‐hydroxyl‐N‐desmethyl‐tamoxifen, and 4‐hydroxy‐tamoxifen [59]. Lower expression of CYP enzymes is associated with unfavourable outcome in ER‐positive patients treated with tamoxifen due to their inability to efficiently metabolise the drug [21]. Similarly, the metabolism of taxol has 19 distinct enzymatic steps, half of which are catalysed by CYP enzymes [60]. Studies report that certain polymorphic forms of CYP enzymes such as CYP3A4 promote the rapid clearance of docetaxel from the bloodstream and lowers efficiency of cyclophosphamide in breast cancer [61]. Furthermore, studies have shown an increase in the transcript levels of *CYP2W1* in responders to NACT compared to breast cancer patients with a stable or a progressive disease [13]. Such studies provide evidence that tumours with lower expression of cytochrome P450 enzymes may not be able to efficiently metabolise certain chemotherapy drugs which may contribute to therapy resistance and impact negatively on patient survival [62]. Current literature supports the findings in the present study that low expression of cytoplasmic CYP2S1 and of nuclear CYP2W1 results in shorter survival of breast cancer patients, a trend which was maintained even in multivariate analysis. Lower expression of CYP2S1 and CYP2W1 was also associated with adverse survival in ER‐positive and ER‐negative patients respectively. The role of these proteins in breast cancer is unclear, but the involvement of cytochrome P450 family members in the metabolism of estrogen and anti‐cancer agents may affect breast cancer prognosis [20, 63]. Future experiments can assess the response of ER‐positive patients to HT, which was not conducted due to lack of treatment information in the current cohort at the time of this study. It should be noted, however, that there was no significant association between *CYP2S1/CYP2W1* mRNA with survival and some of the clinicopathological variables in the METABRIC cohort, compared to that seen in the Nottingham cohort protein assessments. Such discrepancies may reflect the ability of mRNA to undergo extensive post‐transcriptional modifications (e.g. methylation) [64], which may result in a lack of significant correlation between mRNA and translated protein [64]. In addition, mRNA isolated from tissues contains a mixture of tumour, stromal, and immune cells, which may result in dilution effects as opposed to IHC, wherein CYP2S1 and CYP2W1 protein expression was assessed only in tumour cells.

The results from the current study in breast cancer confirm the findings of Ronchi *et al* who reported that, in adrenocortical carcinomas, high CYP2W1 was associated with longer overall survival and time to progression [65]. However, Kumarakulasingham *et al* reported that higher expression of CYP2S1 was associated with poor prognosis in colon cancer patients [34]. Other studies in colon cancer demonstrated that high CYP2W1 was associated with poor survival in grade 2 and 3 colon cancers [14, 66, 67]. A study conducted in 170 breast carcinomas reported that absence of CYP2S1 expression was associated with better survival of breast cancer patients [9]. The reasons for discrepancies observed between the data obtained in the current study and those reported in other tumour types may be due to the differential physiological functions of these proteins in different tissues [62, 68]. Furthermore, the aforementioned findings were conducted in smaller patient cohorts.

In conclusion, the current study demonstrates the prognostic importance of CYP2S1 and CYP2W1 protein expression in both single marker and combined marker analysis. Future studies should assess larger patient cohorts to determine the clinical utility of using CYP2S1 and CYP2W1 as biomarkers, including examining their role in the hormonal and chemotherapeutic response of breast cancer patients.

## Author contributions statement

RAM conducted IHC staining. RAM and SS conducted IHC scoring. RAM, SS and AG performed statistical analyses. ER and IE provided breast tissue and clinical data. SM conceived and funded the study, analysed, and interpreted data. RAM and SM wrote the manuscript. All authors approved the final manuscript for submission.

## Supporting information


**Figure S1.** Kaplan–Meier analysis of the effect of *CYP2S1* and *CYP2W1* mRNA on breast cancer specific survival in the METABRIC cohort
**Table S1.** Correlation of *CYP2S1/CYP2W1* mRNA with clinicopathological parameters in the METABRIC cohort

## Data Availability

Data are available upon request to the corresponding author.
