# Supplementary material for: CYP2S1 and CYP2W1 expression is associated with patient survival in breast cancer
Source: J Pathol Clin Res. 2022 Jul 28;8(6):550–66. doi: 10.1002/cjp2.291 (PMC9535097; doi:10.1002/cjp2.291)
Supplement: Supplementary file 1 — Figure S1. Kaplan–Meier analysis of the effect of CYP2S1 and CYP2W1 mRNA on breast cancer specific survival in the METABRIC cohort Table S1. Correlation of CYP2S1/CYP2W1 mRNA with clinicopathological parameters in the METABRIC cohort [file CJP2-8-550-s001.pdf]

CYP2S1 and CYP2W1 expression is associated with patient survival in breast cancer

R Aiyappa-Maudsley *et al*. *J Pathol Clin Res*, https://doi.org/10.1002/cjp2.291

Supplementary Figure S1 and Table S1

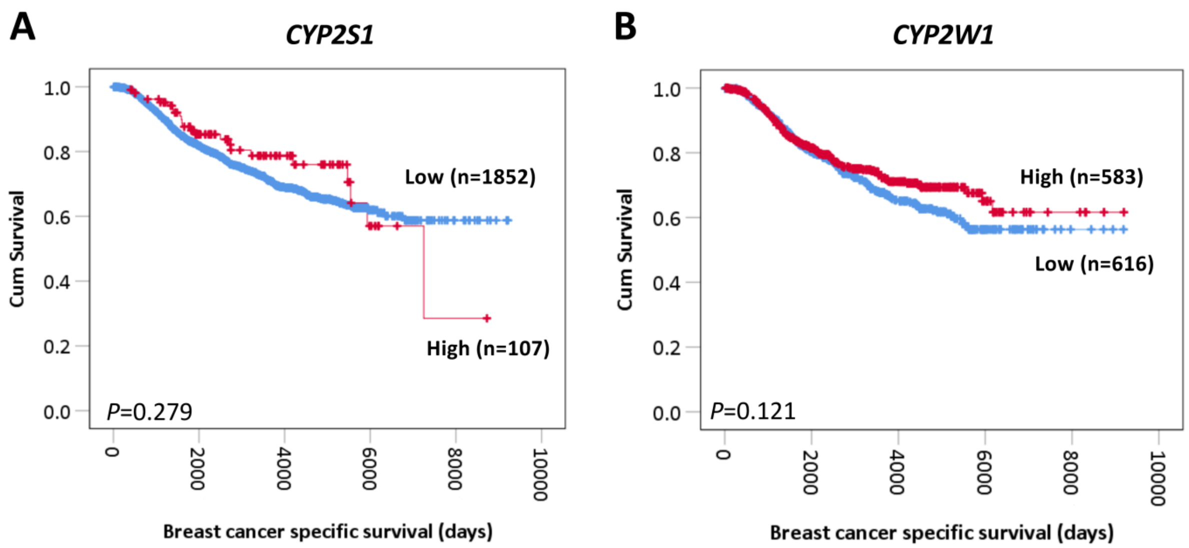

Figure S1. Kaplan-Meier analysis of the effect of *CYP2S1* and *CYP2W1* mRNA on breast cancer specific survival in the METABRIC cohort. Survival curves showing the impact of low (blue line) and high (red line) (A) *CYP2S1* and (B) *CYP2W1* expression on breast cancer specific survival. Significance was determined using the log-rank test. $p > 0.05$ (not significant).

Table S1. Correlation of *CYP2S1/CYP2W1* mRNA with clinicopathological parameters in the METABRIC cohort

**Clinicopathological parameters**	***CYP2S1* mRNA expression**			***CYP2W1* mRNA expression**		
	**Low**	**High**	***P* value**	**Low**	**High**	***P* value**
**Grade**						
1	167 (8.8%)	3 (0.2%)	0.062	60 (5.2%)	51 (4.4%)	0.598
2	733 (38.7%)	37 (2.0%)		245 (21.1%)	221 (19.1%)	
3	895 (47.3%)	57 (3.0%)		291 (25.1%)	291 (25.1%)	
**Size**						
<20 cm	809 (41.4%)	49 (2.5%)	0.69	294 (24.6%)	233 (19.5%)	**0.009**
≥20 cm	1037 (53.1%)	58 (3.0%)		321 (26.9%)	346 (29.0%)	
**Nodal stage**						
1	972 (49.3%)	63 (3.2%)	0.315	338 (28.0%)	298 (24.7%)	0.179
2	595 (30.2%)	27 (1.4%)		196 (16.3%)	180 (14.9%)	
3	299 (15.2%)	17 (0.9%)		88 (7.3%)	105 (8.7%)	
**ER status**						
Negative	442 (22.3%)	32 (1.6%)	0.137	139 (11.5%)	153 (12.6%)	0.136
Positive	1431 (72.3%)	75 (3.8%)		484 (39.9%)	436 (36.0%)	
**PgR status**						
Negative	873(44.1%)	67 (3.4%)	**0.001**	300 (24.8%)	295 (24.3%)	0.502
Positive	1000 (50.5%)	40 (2.0%)		323 (26.7%)	294 (24.3%)	
**HER-2 status**						
Negative	1628 (82.2%)	105 (5.3%)	**0.001**	540 (44.6%)	518 (42.7%)	0.508
Positive	245 (12.4%)	2 (0.1%)		83 (6.8%)	71 (5.9%)	
**Pam50 subtype**						
Basal	298 (15.1%)	31 (1.6%)	**0.009**	88 (7.3%)	112 (9.2%)	**0.02**
HER-2	226 (11.4%)	14 (0.7%)		94 (7.8%)	62 (5.1%)	
Luminal A	691 (34.9%)	27 (1.4%)		228 (18.8%)	198 (16.3%)	
Luminal B	461 (23.3%)	27 (1.4%)		149 (12.3%)	151 (13.1%)	
Normal	191 (9.6%)	8 (0.4%)		63 (5.2%)	54 (4.5%)	

The *P* values are resultant from the Pearson χ^2 test of association. Significant *P* values (<0.05) are indicated in bold. ER is estrogen receptor, PgR is progesterone receptor, and HER-2 is human epidermal growth factor receptor-2. The number and percentage of observations for cohort are shown for each clinicopathological variable.
